# Liver Fibrosis: Interactions Between Cells and Microenvironments

**DOI:** 10.5152/tjg.2025.25313

**Published:** 2025-09-29

**Authors:** Xi Zou, Yunling Ke, Yidan Shao, Shourong Liu, Tingting Shi

**Affiliations:** Department of Pharmaceutical Preparation, The Hangzhou Xixi Hospital Affiliated to Zhejiang Chinese Medical University, Zhejiang, China

**Keywords:** Extracellular matrix stiffness, hepatic stellate cell, liver fibrosis

## Abstract

Liver fibrosis is a key intermediate stage in the progression of chronic liver disease to end-stage liver cirrhosis. Mortality rises exponentially once it reaches decompensated liver disease. In a healthy liver microenvironment, hepatocytes, Kupffer cells, hepatic stellate cells (HSCs), liver sinusoidal endothelial cells, and other cells interact with extracellular matrix (ECM) to maintain cell stability and liver function. Different types of liver injury (such as viral hepatitis and alcoholic liver injury) can cause liver fibrosis. Liver injury signals activate Kupffer cells and recruit immune cells, leading to liver inflammation. This inflammation, together with liver injury, stimulates the activation of HSCs. Activated HSCs migrate to injury sites and secrete ECM. The ECM increase and stiffening contribute to fibrosis. Microenvironment changes alter cell phenotypes, perpetuating HSC activation. This article explores liver fibrosis mechanisms, reviews cellular and microenvironmental changes, summarizes fibrosis characteristics, and provides insights for clinical treatment.

**Main Points**

- Normal liver microenvironment guides the differentiation and maturation of various types of liver cells to produce a wide range of liver functions.
- Injury microenvironment and inflammatory microenvironment are the initial signals of injury repair, but they promote the generation of liver fibrosis in chronic liver injury.
- Activation of hepatic stellate cells by signals in the microenvironment is the key event of liver fibrosis, which will lead to a large number of extracellular matrix (ECM) secretions, and the changes in ECM composition and hardness will induce liver cells to dedifferentiate and lose their normal function.
- Dedifferentiated hepatocytes, liver sinusoidal endothelial cells, and other cells secrete signals to establish the fibrosis microenvironment, which promotes the progression of fibrosis and makes fibrosis irreversible.

## Introduction

**Figure 1.**
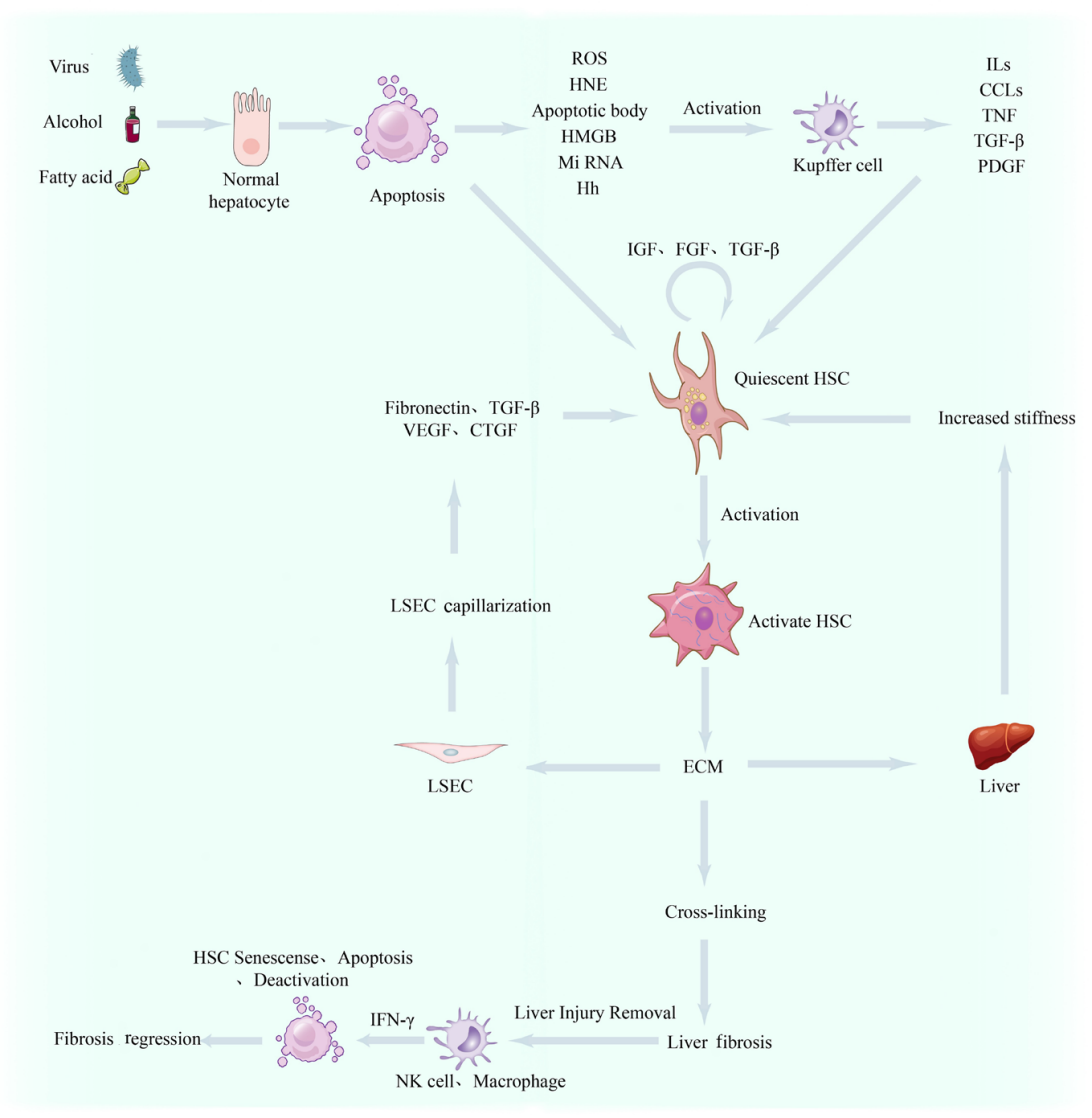
The occurrence and development of liver fibrosis. ROS, Reactive Oxygen Species; HNE, 4-Hydroxynonenal; HMGB, High Mobility Group Box; Hh, Hedgehog; ILs, Interleukins; CCLs ,Chemokine; TNF, Tumor Necrosis Factor; PDGF, Platelet Derived Growth Factor; TGF-β, Transforming Growth Factor-β; IGF, Insulin-like Growth Factor; FGF, Fibroblast Growth Factor; ECM, Extracellular Matrix; LSEC, Liver Sinusoidal Endothelial Cell; VEGF, Vascular Endothelial Growth Factor; CTGF, Connective Tissue Growth Factor; HSC, Hepatic Stellate Cells.

Liver fibrosis is considered a pathological wound-healing response to chronic liver injury. Activated myofibroblasts continuously secrete extracellular matrix (ECM), changing the liver’s microenvironment. Interactions among hepatocytes, hepatic stellate cells (HSCs), liver sinusoidal endothelial cells (LSECs), and resident immune cells (such as Kupffer cells) in the changed microenvironment promote excessive ECM deposition, impair the recovery of parenchymal cells, and result in liver function decompensation (Figure 1).^1^^,^^2^ Most chronic liver injuries can develop into liver fibrosis. These injuries are broadly classified into hepatotoxic and cholestatic injuries based on the site of damage. Hepatotoxic injury refers to hepatocyte death caused by various factors, such as viral hepatitis, metabolic dysfunction–associated steatohepatitis (MASH), and alcoholic liver disease. Cholestatic injury is seen in conditions like primary biliary cholangitis.^3^ Persistent liver injury leads to the fusion of fibrotic segments, contraction, and sclerosis, which distorts the liver’s structure and results in the formation of hepatocyte nodules. This ultimately progresses to cirrhosis, portal hypertension, or liver dysfunction. Liver fibrosis is reversible in early stages, but its reversibility decreases as it progresses to cirrhosis. The global prevalence is estimated to be between 4.5% and 9.0%.^4^ Death is associated with its progression to cirrhosis and hepatocellular carcinoma. Cirrhosis accounts for 2.1% of global mortality, rising to 3.5% after progression to hepatocellular carcinoma.^5^ Around 1.3 million people die from viral hepatitis yearly, and hepatitis B virus patients have decreased by 31.3% due to vaccines. Direct-acting antivirals have led to over a 95% cure rate for hepatitis C virus (HCV), reducing liver fibrosis incidence in some regions. The incidence of liver fibrosis from alcohol and MASH has increased from 25.3% to 38.0% in 10 years, surpassing viral hepatitis.^6^ Current treatments target early-stage fibrosis, and there are no approved treatments for liver fibrosis. This article reviews its mechanisms and aims to find potential drug targets for new treatments.

## Liver Microenvironment Maintains Normal Cellular Phenotype

### The Dynamic Balance of the Normal Liver Microenvironment

The liver microenvironment includes cells and the ECM they secrete. The ECM is primarily composed of collagen types I, III, and IV, as well as laminin and fibronectin, which are structural glycoproteins.^7^^,^^8^ These substances are released by cells into the external environment and not only provide support for cell growth but also act as signals representing the state of cell survival, allowing cells to perform their normal functions. Normally, the ECM density is low and in equilibrium, regulated by matrix metalloproteinases (MMPs) that hydrolyze excess components to reduce resistance for direct contact between liver cells and blood. In acute liver injury, MMPs destroy the ECM, enabling immune cells to repair the liver. After repair, the ECM density normalizes.

### The Role of the Normal Liver Microenvironment

The normal liver microenvironment guides cell differentiation and maturation, enabling liver functions.^9^ This involves integrins on cell surfaces, influencing neighboring cells and mediating cell differentiation. The ECM stiffness and mechanical forces affect the cell phenotype.

**Figure 2.**
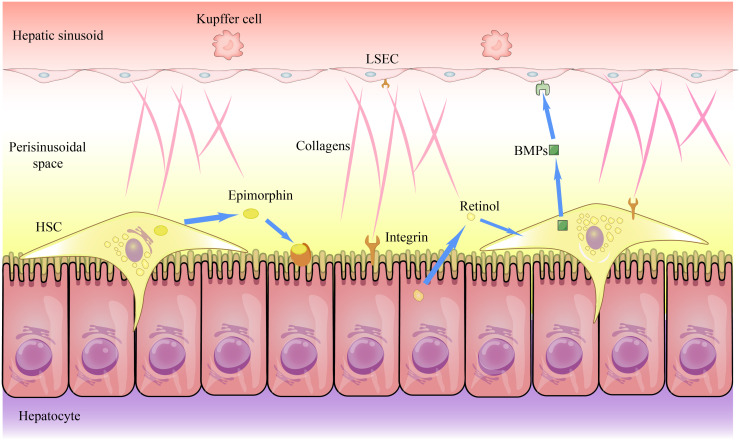
Interactions between liver cells and ECM to maintain normal phenotype and liver function. HSC, Hepatic Stellate Cells; BMPs, Bone Morphogenetic Proteins; LSEC, Liver Sinusoidal Endothelial Cell.

The normal liver microenvironment induces hepatocytes to differentiate for digestive and detoxification functions, and hepatocytes use integrin receptors to interact with the ECM to deliver differentiation signals to the hepatocytes,^10^ generating a hepatocyte phenotype with polarity. Mechanical forces exerted on hepatocytes by ECM can also act as signals that are translated into biochemical signals in the cell to induce hepatocyte differentiation and maturation.^11^ The stiffness of the ECM can affect the phenotype of liver cells. Liver cells in a stiffer matrix show lower differentiation and gain regenerative capacity.^12^ When the matrix returns to normal hardness, they differentiate and mature to perform metabolic functions (Figure 2).

Hepatic stellate cells in the normal hepatic microenvironment are of a quiescent phenotype and release type IV collagen and laminin in a paracrine pathway, which binds to its own integrin receptor and promotes HSC differentiation to a quiescent phenotype. In a quiescent state, HSCs extend tentacles to contact hepatocytes for signal transmission. Hepatocytes secrete retinol into HSCs to maintain their quiescent phenotype.

The LSECs interact not only with ECM components but also with blood, and their integrin receptors sense changes in blood shear stress.^13^ These signals are mediated within the cells via the transcription factor Kruppel-like factor 2, maintaining the normal phenotype of LSECs.^14^ Bone morphogenetic proteins (BMPs) secreted by HSCs play an important role in maintaining LSEC quiescence.^15^ Growth factors such as vascular endothelial growth factor (VEGF) regulate the phenotype of LSECs.^16^

## Injured Microenvironment Directs Immune Cells to Establish an Inflammatory Microenvironment

### Injured Microenvironment and Inflammatory Microenvironment

**Figure 3.**
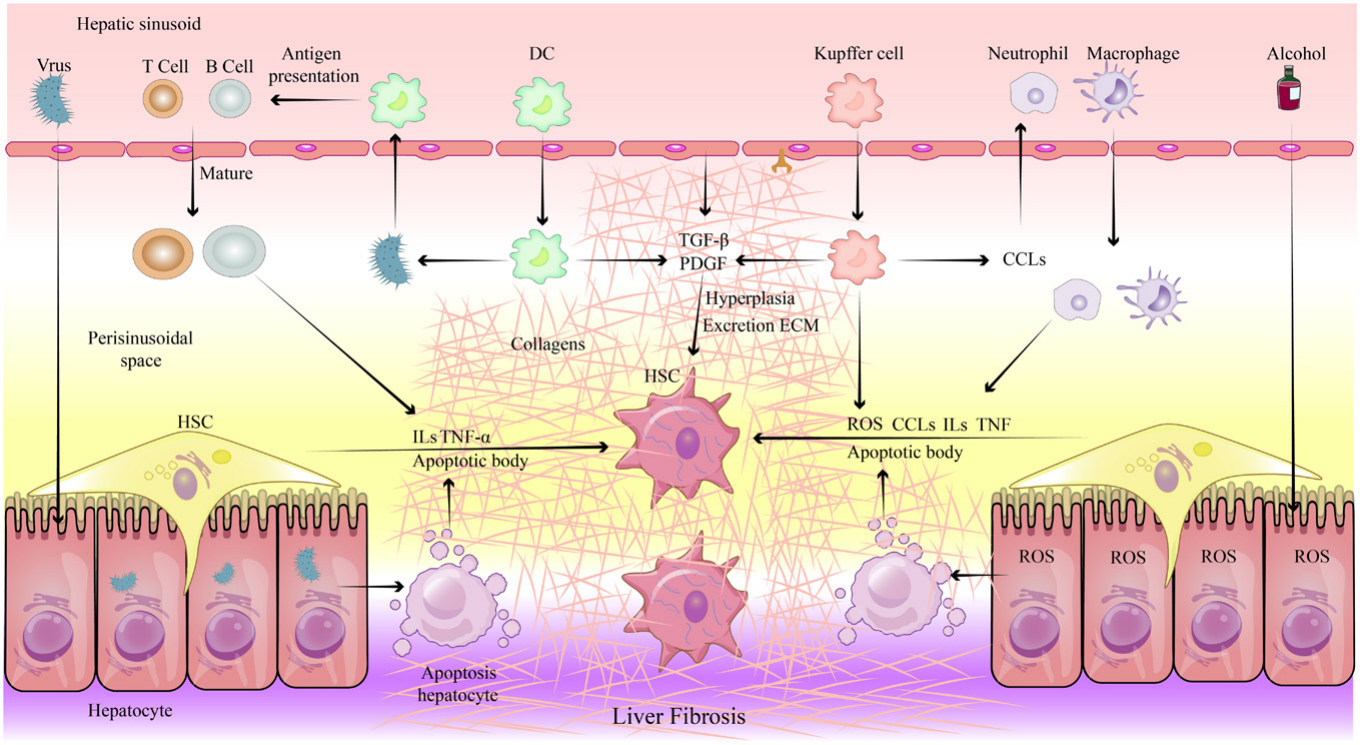
Inflammation and HSC activation following liver injury. IFN, Interferon; TGF-β, Transforming Growth Factor-β; PDGF, Platelet-Derived Growth Factor; HSC, Hepatic Stellate Cells; DC, Dendritic Cells; ILs, Interleukins; CCLs, Chemokine; TNF, Tumor Necrosis Factor; ROS, Reactive Oxygen Species.

When the liver is damaged, an injured microenvironment forms as cells release substances, including immunogenic ones, into the surroundings. These act as signaling molecules to transmit danger information to nearby cells, guiding the liver’s stress response. Resident immune cells are the first to act. Once the danger signal is released, they quickly reach the damaged area to establish an inflammatory microenvironment for immune cell activation. This microenvironment, characterized by immune cell released cytokines and reactive oxygen species (ROS), is key to liver fibrosis development (Figure 3).

### The Injured Microenvironment Influences the Development of the Inflammatory Microenvironment

Liver injury can be pathogen-mediated or metabolic, and the release of cellular contents as well as immunogenic substances from injured hepatocytes into the microenvironment is key to the activation of inflammation.^17^ Although damage types vary,^18^^,^^19^ these contents activate the immune response using injury-associated molecular patterns and pathogen-associated molecular patterns. Kupffer cells recognize signals released by damaged hepatocytes through Toll-like receptors 4 (TLR4), activating Nuclear Factor-κB (NF-κB) and inducing the expression of inflammatory cytokines such as tumor necrosis factor-α (TNF-α) and interleukins (IL-6 and IL-1β). This recruits bone marrow–derived immune cells, initiating an inflammatory cascade (Figure 3). Recruited immune cells include macrophages, classified as pro-inflammatory (M1) or anti-inflammatory (M2). M1 macrophages secrete inflammatory cytokines (IL-6, TNF-α, IL-1) and recruit other immune cells while activating HSCs to direct fibrosis production.^20^ During the later stages of fibrosis, M2 macrophages are recruited to promote collagen dissolution through the secretion of MMP9 and MMP12^21^ and facilitate tissue repair by releasing IL-10, transforming growth factor-β (TGF-β), and VEGF, and they could promote M1 macrophage apoptosis to inhibit inflammation. After injury, neutrophils are quickly recruited to clear damaged cells and secrete ROS, TNF-α, and IL-1 promoting fibrosis. Liver-present dendritic cells are activated by immunogenic substances, triggering an adaptive immune response. T helper 17 (Th17) cells and natural killer T (NKT) cells are then recruited to the liver, where they secrete IL-4 and IL-13. These are pro-fibrogenic factors that accelerate the process of fibrosis.^22^ Both humoral and cellular immunity contribute to fibrosis progression (Figure 3). The injured and inflammatory microenvironments are key factors in activating liver fibrosis.

## Different Microenvironments Guide Hepatic Stellate Cell Activation

### The Role and Origin of Myofibroblasts

The key step in fibrosis is the activation of HSCs. Hepatic stellate cell activation refers to the process by which HSCs transition from a quiescent phenotype to myofibroblasts during liver injury. During this transition, they acquire capabilities for proliferation, fibrogenesis, inflammation, contraction, and migration.^17^ Activated HSCs display an increased number of membrane surface receptors and, under the influence of growth factors and chemokines, migrate to and proliferate at the site of injury. They secrete large amounts of ECM, particularly types I and III collagen. This process, mediated by factors such as MMPs and endothelin, leads to microenvironment remodeling, and the sustained activation of HSCs will establish the fibrotic microenvironment. The source and composition of myofibroblasts vary significantly depending on the cause of liver fibrosis. Regardless of the type of liver injury, HSCs are the primary source of myofibroblasts.^23^ In addition to resident cell differentiation, various bone marrow-derived cells contribute to fibrosis during the middle and late stages of injury. Circulating fibrocytes reach the liver via the bloodstream and differentiate into myofibroblasts, promoting the progression of fibrosis.^24^

### Inflammatory Microenvironment Induces Hepatic Stellate Cell Activation

Growth factor signaling is crucial for the activation of HSCs, with key factors including TGF-β, platelet-derived growth factor (PDGF), and VEGF.

**Figure 4.**
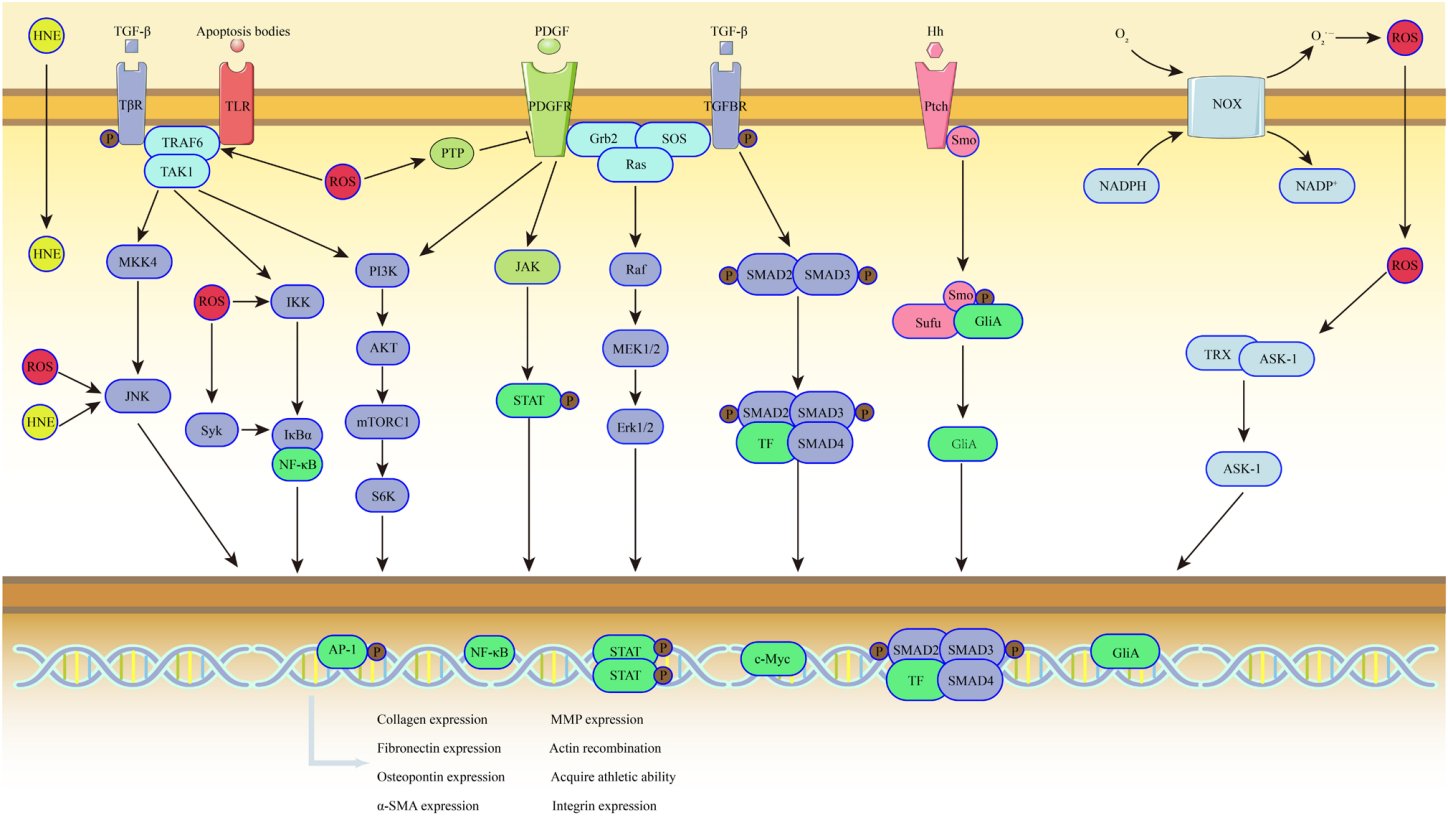
Intracellular signals related to HSC activation. HNE, 4-Hydroxynonenal; ROS, Reactive Oxygen Species; TGFBR, TGF-β receptor; TRAF6, Tumor Necrosis Factor Receptor-Associated Factor 6; TAK1, TGF-β Activated Kinase 1; MKK4, MAP kinase kinases 4; JNK, C-Jun Amino Terminal Kinase; IKK, IκB kinase; NF-κB, Nuclear Factor-κB; IκB, Inhibitor of NF-kB; PI3K, Phosphatidylinositol-3 kinase; mTORC1, mTOR complex 1; S6K, S6 kinase; Grb2, Growth Factor Receptor-Bound Protein 2; Erk1/2, Extracellular Signal Regulated kinases 1/2; Hh, Hedgehog; Ptch, Patched Receptor; Smo, Smoothened Receptor; Sufu, Suppressor of Fused Homolog; GliA, Glioma-associated oncogene A; TLR, Toll like receptors; PTP, Phosphatases; MEK1/2, Mitogenactivated and extracellular signal-regulated kinase kinase1/2; SOS, Son-of-Sevenless; TF, Transcription factors; NOX, NADPH oxidase; TRX, The redox protein thioredoxin; ASK-1, Apoptosis signal-regulating kinase-1.

Hydrolysis of TGF-β precursors in the inflammatory microenvironment initiates HSC activation. Recruited bone marrow–derived macrophages significantly express higher levels of TGF-β. The TGF-β is stored in the ECM in a latent form, bound to latent TGF-β-binding protein, forming a large latent complex.^25^ The TGF-β hydrolases are secreted by macrophages into the microenvironment, such as urokinase, kinin-releasing enzyme, and fibrinolytic enzymes, which are coupled with exposure of hydrolysis sites to promote TGF-β activation.^26^ Activated TGF-β binds to the TGF-β receptor on HSCs and regulates the transcription of multiple target genes, including α-smooth muscle actin (α-SMA) and connective tissue growth factor (CTGF). This marks the initiation of HSC activation (Figure 4). Activated HSCs express a large number of growth factor receptors in preparation for further activation and proliferation.

The PDGF, secreted by platelets and LSECs, promotes the proliferation, survival, migration, cytoskeletal rearrangement, and actin filament system reorganization of HSC. It also stimulates ECM synthesis (Figure 4).^27^ Upon activation of HSC by TGF-β, the expression of PDGF receptor on the cell membrane is increased, and the massive proliferation after receiving PDGF signals leads to a sustained rise in the number of HSCs at the site of injury.^28^ In addition, CTGF is a potent fibrogenic cytokine that contributes to ECM production as well as promotes cell proliferation, migration, adhesion, and survival. The VEGF, secreted by activated HSCs and LSECs, mainly promotes angiogenesis after liver injury and can also induce HSC proliferation.^29^

Activated HSCs and macrophages form tight connections via cadherin-11, enabling macrophages to secrete TGF-β to HSCs directly. This results in prolonged HSC activation.^30^ Neutrophils and mast cells primarily secrete IL-17 and IL-22, which enhance TGF-β signaling in HSCs and further promote their activation. The NKT cells are recruited to the injury site, where they promote liver fibrosis by producing IL-4, IL-13, Hedgehog (Hh), and osteopontin. The IL-13, secreted by Th2 cells, can directly induce the expression of type I collagen and α-SMA proteins, promoting HSC activation.^31^ Activated HSCs recruit inflammatory cells through the production of chemokines and adhesion molecules. Additionally, HSCs present antigens to T lymphocytes and NKTs, perpetuating inflammation and driving fibrosis progression.

Immune cells destroy the normal liver microenvironment by secreted MMPs, and directs the LSECs phenotypic changes, allowing LSECs to lose their original filtering function and gain the ability to secrete new ECM. The secretion of fibronectin by LSECs at the injury site is crucial to HSC activation. Fibronectin interacts with the integrin α9β1 receptor on HSC surfaces, promoting their migration to the injury site and mediating chemotaxis.^32^ The fibronectin expressed by LSECs during fibrosis is primarily the ED-A splice variant, which binds to integrins, generating mechanical tension. This tension can release the hydrolytic site of Pro-TGF-β embedded in the ECM, promoting the activation of TGF-β.^33^ Additionally, LSECs during fibrosis release TGF-β along with Pro-TGF-β hydrolases such as urokinase, kallikrein, and plasmin, promoting HSC activation and fibrosis. Exosomes secreted by LSECs contain high levels of sphingosine kinase 1 (SK-1), which promotes HSC migration and activation.^34^

### Injury Microenvironment-Mediated Hepatic Stellate Cell Activation

**Figure 5.**
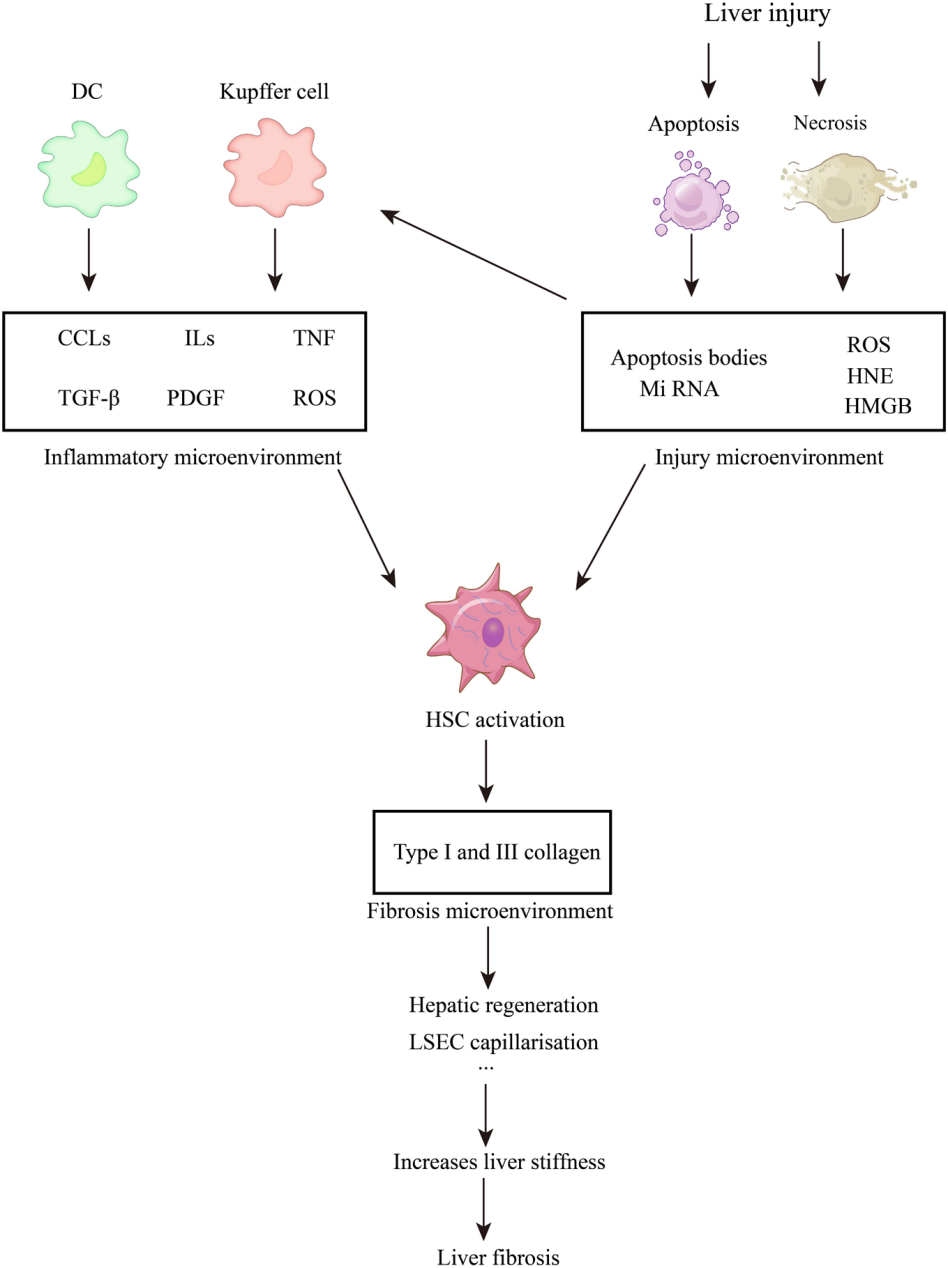
Injury microenvironment and inflammatory microenvironment lead to fibrosis microenvironment. ILs, Interleukins; CCLs, Chemokine; TNF, Tumor Necrosis Factor; HMGB, High Mobility Group Box; TGF-β, Transforming Growth Factor-β; PDGF, Platelet-Derived Growth Factor; ROS, Reactive Oxygen Species; HNE, 4-Hydroxynonenal; LSEC, Liver Sinusoidal Endothelial Cell.

After hepatocyte injury, large amounts of substances are released, with the most critical factors related to HSC activation being ROS, lipid peroxides (such as 4-hydroxy-2,3-nonenal, HNE), and apoptotic bodies (Figure 5).^35^

Although the factors leading to hepatocyte injury vary across chronic liver diseases, most of these inducers can stimulate the generation of ROS and HNE, which are produced intracellularly and released into the microenvironment with hepatocyte necrosis. In alcoholic liver disease, ROS generation involves the mitochondrial respiratory chain and the metabolism of ethanol by cytochrome P450 2E1 (CYP2E1).^36^ In MASH, mitochondrial dysfunction mediates ROS production. After chronic HCV infection, viral core proteins may induce mitochondrial damage in hepatocytes, increasing ROS production.^37^ In addition to the intracellular ROS, activated immune cells and HSCs themselves produce ROS. Furthermore, the liver’s active fat metabolism triggers lipid peroxidation, resulting in the production of aldehyde substances like HNE, which play a crucial role in stimulating collagen expression by HSCs.^38^

A significant portion of highly reactive ROS, such as superoxide anions, produced by the body is hydrolyzed into H_2_O_2_ by superoxide dismutase, reducing its reactivity. Combined with the cell’s own antioxidant systems that metabolize H_2_O_2_, the body can resist naturally occurring ROS. However, certain metal ions can upregulate ROS reactivity. In the Fenton reaction, iron reacts with H_2_O_2_ to produce iron ions, hydroxyl radicals, and hydroxyl free radicals. The H_2_O_2_ is converted into highly reactive hydroxyl free radicals or iron ions, thereby exacerbating oxidative damage. Iron typically binds to iron-regulatory proteins (IRPs), which regulate iron homeostasis through interactions with iron-responsive elements. The ROS modify IRPs, reducing intracellular iron to prevent further iron-mediated oxidative damage. However, this balance is disrupted when ROS are continuously generated. Consequently, fibrosis becomes significant when liver iron levels exceed 60 mmol/g of dry weight.^39^ During liver fibrosis, the Fenton reaction promotes HSC activation. Copper is another catalyst for ROS. Wilson’s disease is a good example. It is a hereditary disorder affecting copper metabolism. The absence of the copper ion transporter protein causes copper ions to accumulate in the liver. This copper overload leads to a significant increase in ROS production.^40^

The ROS regulate HSC activation by enhancing the interaction between cytokines and their receptors (Figure 4). One major way ROS upregulate growth factor signaling is by enhancing receptor tyrosine kinase (RTK) activation. Protein tyrosine phosphatases (PTPs) typically dephosphorylate RTK receptors to shut down the signaling.^41^ The ROS act on the cysteine residues in the active site of PTPs, modifying them into sulfinic acid, which inactivates PTPs. Under physiological conditions, sulfinic acid modifications are rapidly neutralized by reductants. However, in the presence of significant intracellular ROS, these modifications convert into sulfonic acid modifications, resulting in irreversible changes. This leads to prolonged blocking of PTP-dependent receptor dephosphorylation, which allows for enhanced downstream signaling of RTKs. Besides regulating receptor activity, downstream signaling can also affect HSC activation, with NF-κB and the activator protein 1 (AP-1) being the most critical. NF-κB can promote the activation of HSCs and plays a crucial role in the development of liver fibrosis. Reactive oxygen species activate the kinase Syk, which phosphorylates tyrosine 42 on inhibitor of NF-kB α (IκBα), facilitating its degradation.^42^ The ROS also modify tumor necrosis factor receptor-associated factor 6, which forms a complex with receptors, subsequently promoting NF-κB activation. The AP-1, a dimeric transcription factor composed of c-Jun and c-Fos, can be activated by low levels of ROS. Intracellular ROS induce the dimerization of thioredoxin, promoting the dissociation of apoptosis signal-regulating kinase-1, which ultimately mediates HSC activation through AP-1 phosphorylation. Reactive oxygen species can activate HSCs through non-growth factor mechanisms. For instance, ROS produced by HSCs can enhance protein kinase activity, leading to RTK activation in a ligand-independent manner.^43^ After entering HSCs, ROS can activate cyclooxygenase-2 (COX-2) or redox-sensitive transcription factor Sp-1, which mediates the upregulation of collagen expression and promotes fibrosis progression.

As the number of patients with MASH continues to rise, it has been found that HNE has significant harmful effects on cells, especially in liver fibrosis. The HNE easily crosses HSC membranes, leading to increased type I collagen synthesis and promoting fibrosis progression.^44^ It enhances the binding of C/EBPβ, a downstream signaling protein of TGF-β, to specific regions of the type I collagen α1 gene promoter, thereby increasing type I procollagen expression. Furthermore, HNE activates JNK upon contact with HSCs, which increases AP-1 binding to DNA. Lipid peroxides mainly promote fibrosis progression by stimulating HSC secretion of ECM. Apoptotic bodies can activate HSCs by generating ROS (Figure 4). Studies indicate that apoptotic bodies interact with HSCs TLR9 receptors, mediating their phagocytosis and inducing NADPH oxidase (NOX) expression. The NOX-generated ROS further stimulate HSC activation.^45^

Genetic studies have shown that the Hh signaling pathway plays a key role in cell morphogenesis and regulates the fate of liver progenitor cells, promoting their differentiation into mature hepatocytes during normal liver cell turnover. During fibrosis, Hh signaling activates HSCs and promotes the secretion of osteopontin (Figure 4). Hedgehog is released by damaged hepatocytes. For instance, in MASH, endoplasmic reticulum stress in hepatocytes induces Hh expression, which then signals externally, leading to HSC activation. The Hh signaling facilitates the binding of the transcription factor Gli2 to target genes, controlling cell survival, proliferation, migration, and differentiation.^46^ Additionally, Hh signaling activates the Hippo/Yes-associated protein 1 (Yap1) pathway to promote myofibroblast morphogenesis.

## Construction of a Fibrotic Microenvironment

Under the influence of injured and inflammatory microenvironments, HSCs are activated in large numbers, leading to the formation of a fibrotic microenvironment characterized by ECM secretion by activated HSCs.

Hepatocytes, Kupffer cells, and activated HSCs themselves secrete chemokines (CCLs) at the injury site, such as CCL2, CCL5, and CXCL8, which promote nearby HSC activation and migration to the injury site. These chemotactic signals mediate cytoskeletal remodeling in HSCs, resulting in actin production at HSC projections. Actin contraction facilitates HSC movement toward the injury site. The interaction between fibronectin and integrin receptor α9β1 generates mechanical forces that promote the migration of HSCs to the site of injury.^32^ The PDGF enhances HSC migration, promoting the movement of activated HSCs to the injury site. With increasing chemotactic signals, a large number of HSCs migrate to the injury site, where they align and secrete ECM, leading to the formation of fibrous septa.

Chronic liver injury causes persistent ECM deposition. The TGF-β and CTGF stimulate HSCs to secrete large amounts of ECM components. As their quantity increases, the solubility of the ECM decreases, resulting in its precipitation and the formation of fibrotic scar tissue. Excessive ECM production causes cross-linking, making it insoluble and resistant to hydrolysis, and difficult for MMPs to degrade. The fibrosis microenvironment is characterized by changes in ECM composition and an increase in quantity, resulting in ECM hardening. Different types of liver injuries lead to ECM deposition in distinct areas. For example, viral liver diseases primarily result in ECM deposition around the portal vein, whereas fatty liver disease leads to ECM accumulation in the centrilobular region. As HSC activation continues, ECM production expands, causing cross-linking and fusion of adjacent regions, creating fibrotic bridging. Once established, the fibrosis microenvironment promotes fibrosis.

Activated HSCs express α-SMA, SMA, and myosin, which are markers of contractility in non-muscle cells. Activated HSCs upregulate the expression of endothelin-1 (ET-1), vasopressin, and angiotensin II (Ang-II) receptors, promoting HSC contraction.^47^ In a healthy liver, ET-1 is produced by LSECs, but during liver fibrosis, HSCs become the primary source of ET-1 as LSEC secretion decreases. The HSC contraction affects fibrosis by regulating liver blood flow and ECM remodeling, thereby promoting fibrosis. In a healthy liver, HSCs are located in the perisinusoidal space, with long projections that encircle the sinusoids. During fibrosis, contraction regulates sinusoidal resistance and blood flow. The ET-1 and Ang-II promote HSC contraction, leading to portal hypertension. Hepatic stellate cell contraction can remodel the ECM, drawing scattered ECM components together and promoting ECM cross-linking, leading to the formation of septa and fibrotic bridging.

## Fibrosis Microenvironment Promotes Disease Progression

### Fibrosis Microenvironment Increases Liver Stiffness and Promotes Fibrosis Development

Activated HSCs increase ECM secretion, and a critical change: the increased stiffness of the ECM, a physical property that promotes the progression of fibrosis to cirrhosis. Extracellular matrix stiffness is primarily regulated by several enzymes released by HSCs. Lysyl oxidase (LOX) is particularly associated with the progression of fibrosis, and its levels are significantly elevated in the serum of fibrotic patients.^48^ Lysyl oxidase mediates collagen cross-linking, promoting local adhesion and ECM deposition, which in turn increases tissue stiffness. Lysyl oxidase catalyzes the oxidation of lysine residues in collagen into peptidyl aldehydes, which spontaneously condense with nearby peptidyl aldehydes, leading to the covalent cross-linking of collagen and elastin. This process reduces the solubility of ECM proteins, making them more stable. Type I collagen is LOX’s primary target. After LOX modifies type I collagen, fiber cross-links form between molecular chains, increasing the mechanical properties of the fibers.^49^ Lysyl oxidase is typically secreted in an inactive precursor form. The BMP-1 produced by TGF-β-activated HSCs cleaves pro-LOX into its active form, promoting ECM stiffening.^50^ Transglutaminase (TG) is another key enzyme that regulates ECM stiffness. The TG catalyzes covalent cross-links between glutamine and lysine residues in collagen and fibronectin, providing additional stiffness and resistance to proteolysis.^51^ Fibronectin further mediates collagen assembly after fiber deposition, promoting ECM stiffening.

Changes in the physical properties of the microenvironment significantly impact fibrosis. Culturing hepatocytes in a soft ECM environment allows them to maintain normal function for an extended period. However, in a stiffened microenvironment, hepatocyte metabolic activity and protein secretion capacity decrease. This loss of function is primarily regulated by hepatocyte nuclear factor 4α (HNF4α).^52^ The persistence of this stiffened physical environment leads to hepatocyte senescence and loss of regenerative capacity, which may explain why liver parenchyma fails to regenerate after cirrhosis.

### Fibrosis Microenvironment Promotes Liver Sinusoidal Endothelial Cell Capillarization

Fibrosis microenvironment directs dramatic changes in LSECs phenotype and function. The increase in ECM serves as one source of these signals. Excessive ECM forms a basement membrane-like structure in the space of Disse, inducing LSEC phenotypic changes into normal capillaries—a process called capillarization. During this process, LSECs lose their original filtering and endocytic functions. Changes in ECM composition also act as signaling factors. Excessive production of type I collagen directly alters LSEC fenestrations, while increased laminin concentration causes the disappearance of endothelial pores. The ECM interacts with integrin receptors on LSECs, modifying their phenotype. In a healthy liver, only 2 types of integrin receptors—α1β1 and α5β1—are present on LSEC surfaces. However, laminin production increases integrin α6β1 expression on LSEC surfaces, mediating phenotypic changes through interactions with ECM. A stiffened microenvironment promotes LSEC capillarization, but if stiffness decreases, the phenotype can recover. In fibrosis, Hh ligands secreted by macrophages and HSCs induce LSEC capillarization.^53^ Changes in shear stress also play a role in mediating LSEC phenotypic changes. Under physiological shear stress, NO production regulates vascular size and maintains LSEC phenotype. However, during fibrosis, the synthesis of NO by LSECs decreases, resulting in increased shear stress. As the shear stress increases, the phenotype of LSECs changes accordingly.

After the phenotypic changes in LSECs, their functions are altered, promoting the progression of fibrosis. Following liver fibrosis, LSECs’ immune function shifts from a tolerogenic to a pro-inflammatory state. Capillarized LSECs secrete a series of cytokines (e.g., TNF-α, IL-6, IL-1, and CCL2) that enhance inflammatory responses, increase immune cell chemotaxis, capture antigens, and induce T cell proliferation.^54^ The LSECs upregulate fibronectin EIIIA fragment expression, mediating immune cell adhesion. Fibronectin EIIIA can also activate HSCs,^33^ further promoting fibrosis. During fibrosis, LSECs overproduce kallikrein and plasmin, which hydrolyze latent TGF-β, exacerbating liver fibrosis.^55^ The TGF-β acts on LSECs to mediate epithelial-mesenchymal transition, promoting myofibroblast formation. During fibrosis, reduced NO production and increased endothelin-1 expression in LSECs contribute to portal hypertension.

## Conclusion

This article aims to review the main mechanisms of the liver fibrosis microenvironment, offering insights for new therapeutic approaches and advancing basic research toward clinical applications. Currently, there is no particularly effective treatment for liver fibrosis. The main focus is on reducing liver injury and inflammation. Activating peroxisome proliferator-activated receptors-γ (PPARγ) improves fat deposition in the liver, relieves insulin resistance, and reduces de novo lipogenesis in the liver. Clinical trials have demonstrated the effectiveness of PPARγ agonists in treating MASH-related liver fibrosis.^56^ The PPARγ can induce HSC inactivation. This means that it targets not only liver damage but also has a direct inhibitory effect on liver fibrosis. The therapeutic effects of current PPARγ agonists in treating liver fibrosis caused by other types of liver injury are unknown. The transplantation of stem cells has received considerable attention in various clinical trials for liver fibrosis. Although stem cell transplantation therapy can promote liver regeneration and suppress inflammation, its carcinogenic properties increase the uncertainty surrounding the treatment. A key issue for the future is how to increase and maintain an effective concentration of stem cells in the liver.^57^

There are currently no approved methods of antifibrotic treatment. The most direct way to reverse fibrosis is to promote ECM degradation. Using MMP-9-derived recombinant mutant proteins to inhibit tissue inhibitors of metalloproteinases-1 (TIMP-1) enhances MMP activity, thereby promoting ECM hydrolysis. Although MMPs are more active, the increased hardness of the ECM makes it significantly more resistant to them. Selective inhibition of LOX2 can reduce ECM cross-linking and promote ECM hydrolysis. A drug that acts on both ECM hardness and MMPs could become the mainstay of antifibrotic treatment. Using inactivated HSCs to promote the regression of liver fibrosis is a new treatment approach. Hypoxia-inducible factor 1α regulates the energy metabolism of HSCs and induces their inactivation. The mechanism of HSCs inactivation is currently unclear, and inducing HSCs inactivation is an important future development trend.^58^

The ability to reverse liver fibrosis decreases as the condition worsens. Therefore, it is important to identify it in the early stages of fibrosis. The most common clinical diagnostic method for predicting the risk of liver fibrosis is to combine the results of transaminase and platelet count tests with the patient’s age. Some gene products can also act as biomarkers for liver fibrosis. These include non-coding RNAs, which are significantly associated with the severity of liver fibrosis. Recent studies on the relationship between liver fibrosis and changes in the gut microbiome have shown that the diversity of bacterial communities varies significantly at different stages of fibrosis. Bacterial communities can currently be used to accurately identify liver cirrhosis, and it is possible that, in the future, the gut microbiome may also become a marker of liver fibrosis.^59^

In addition to blood serum biomarkers, imaging technology plays a key role in identifying liver fibrosis. This technology mainly detects liver elasticity, as ECM hardness is closely linked to liver fibrosis. Elastography (LSM), when combined with ultrasound and magnetic resonance imaging, can be used to assess liver stiffness in real time. Vibration-controlled transient elastography was the first LSM program introduced. It offers fast, accurate detection and can be performed bedside. However, it has a certain failure rate, and combining it with serum biomarkers can provide more accurate conclusions. Magnetic resonance elastography can also assess tissue elasticity and stiffness, outperforming transient elastography. Still, elastography may fail or produce unreliable results in obese or ascitic patients. The advantage of imaging techniques in chronic liver disease diagnosis lies not only in their diagnostic capabilities but also in monitoring liver fibrosis progression. Although failure rates exist, standardized procedures and experienced operators can minimize them, making imaging techniques one of the major diagnostic methods for liver fibrosis in the future.

## Data Availability

The data that support the findings of this study are available on request from the corresponding author.
